# Application of injectable hydrogels in the treatment of periodontitis

**DOI:** 10.3389/fbioe.2026.1874053

**Published:** 2026-07-09

**Authors:** Jiayi Sun, Mingtong Wu, Sa Bao, Tianqi Shen, Lihua Luo, Yuannan Sun

**Affiliations:** 1 School and Hospital of Stomatology, Wenzhou Medical University, Wenzhou, Zhejiang, China; 2 Department of Pharmacy, Affiliated Xiaoshan Hospital, Hangzhou Normal University, Hangzhou, China

**Keywords:** injectable hydrogels, local drug delivery, periodontitis, stimuli-responsive, tissue regeneration

## Abstract

Periodontitis is a chronic inflammatory disease driven by dental plaque biofilms and characterized by chronic inflammation, oxidative stress, and progressive alveolar bone destruction. Although conventional therapies can control infection, their clinical efficacy is often limited by insufficient local drug retention, short release duration, and limited regenerative capacity. Injectable hydrogels have emerged as promising local delivery systems because of their biocompatibility, injectability, and controllable release behavior, allowing minimally invasive administration into irregular periodontal defects. Beyond prolonged local drug delivery, injectable hydrogels can also modulate inflammation and promote periodontal tissue regeneration through combined antibacterial, antioxidative, and immunoregulatory effects. This review summarizes recent advances in injectable hydrogels for periodontitis treatment, with emphasis on material classification, multifunctional therapeutic strategies, and stimuli-responsive systems triggered by pH, reactive oxygen species, and temperature changes. Remaining challenges related to mechanical stability, functional integration, and clinical translation are also highlighted.

## 1 Introduction

Periodontitis is a chronic multifactorial inflammatory disease associated with dysbiotic dental plaque biofilms. It is characterized by the progressive destruction of periodontal supporting tissues, such as gingiva, and alveolar bone, ultimately causing tooth mobility and loss (Amato et al., 2022). It is estimated that over one billion people worldwide are affected, with a particularly high prevalence among older adults (Shan et al., 2025; Tao et al., 2025). Beyond negatively affecting oral health and quality of life, periodontitis is associated with systemic diseases, including diabetes, and Alzheimer’s disease (Shan et al., 2025; Tao et al., 2025).

Conventional therapeutic strategies primarily involve mechanical debridement, such as scaling and root planing (SRP), and systemic or local administration of antibiotics (Khademi et al., 2025). However, mechanical debridement alone is often inadequate for completely removing biofilms from complex periodontal pockets, with limited capacity for regenerating alveolar bone defects. Moreover, long-term or high-dose antibiotic usage may induce antimicrobial resistance and disrupt oral microbiota homeostasis, thereby reducing therapeutic efficacy (Tao et al., 2025). Therefore, the development of more targeted, efficient, and safer therapeutic strategies is critically needed. Advances in biomaterial sciences and regenerative medicine have increasingly emphasized modifying the periodontal microenvironment and enhancing tissue regeneration rather than solely employing antibacterial treatments (Shan et al., 2025; Tao et al., 2025). Recent studies on bioactive materials in tissue repair have highlighted the importance of simultaneously regulating inflammation, oxidative stress, angiogenesis, and tissue regeneration within chronic pathological microenvironments (Yang et al., 2025). The simultaneous delivery of bioactive substances such as antimicrobial agents and extracellular vesicles effectively mitigates inflammation and promotes tissue healing (El-Sherbiny and Yacoub, 2013; Pan Q. et al., 2025). Nevertheless, clinical translation remains limited by challenges including rapid clearance, poor stability, and difficulties in achieving sustained and precise release (Da Rocha et al., 2015; Pan Q. et al., 2025). Therefore, research has increasingly focused on developing localized delivery platforms with enhanced encapsulation efficacy and controlled release dynamics.

Hydrogels, as cross-linked polymeric networks rich in water, structurally resemble the extracellular matrix (ECM), creating a conducive environment for cell proliferation. They also facilitate the local delivery and sustained release of therapeutic agents (Li Q. et al., 2024; Zhang et al., 2024). Given these advantageous characteristics, hydrogels have become prominent drug delivery systems in periodontal regeneration therapies (Li Q. et al., 2024; Zhang et al., 2024; Dal-Fabbro et al., 2025; Wang S. et al., 2026). Based on delivery methods and gelation mechanisms, hydrogels are categorized into pre-formed and injectable types. Pre-formed hydrogels are fabricated *ex situ* and implanted at target sites via surgical procedures. Injectable hydrogels, however, allow minimally invasive delivery through injection. Recent advances in material science have enabled hydrogel precursors to be administered via syringe, subsequently undergoing *in situ* gelation through physical or chemical crosslinking at the target sites (Dimatteo et al., 2018). Compared with pre-formed materials, injectable hydrogels exhibit superior adaptability to complex tissue architectures, making them particularly suitable for filling irregular defects (Liu et al., 2017; Zhu et al., 2022). Injectable hydrogels facilitate minimally invasive administration into deep periodontal pockets, closely adapting to surrounding tissues and reducing surgical trauma (Ma et al., 2024; Zhang et al., 2024; Zhou et al., 2026). Therefore, injectable hydrogels demonstrate significant potential for the localized and precise treatment of periodontitis (Li Q. et al., 2024; Zhao et al., 2025; Wang S. et al., 2026).

This review systematically summarizes recent advances in injectable hydrogels for treating periodontitis. First, their key physicochemical properties and potential advantages are outlined. Injectable hydrogels are then classified based on polymer origin, and applications involving natural, synthetic, and composite injectable polymeric hydrogels in periodontitis treatment are presented. Moreover, injectable hydrogels responsive to various stimuli are examined for their ability to adaptively respond to pathological alterations in the periodontal microenvironment, including shifts in pH, temperature, ROS levels, enzyme activity, and external triggers such as light. Finally, existing limitations and prospective research directions are discussed to provide both theoretical insights and a practical framework for progressing injectable hydrogels from experimental stages to clinical application.

## 2 Pathogenesis of periodontitis

### 2.1 Bacteria-mediated inflammatory responses and signaling pathway activation

Periodontitis primarily arises due to biofilm accumulation and colonization by pathogenic microorganisms, especially Gram-negative anaerobic bacteria such as Porphyromonas gingivalis (P. gingivalis) and *Fusobacterium* nucleatum (F. nucleatum) (Xi et al., 2019; Ma G. et al., 2025; Li et al., 2026a). These pathogens secrete virulence factors, including lipopolysaccharides (LPS) and proteolytic enzymes, which initiate host immune reactions, trigger inflammatory signaling pathways, and provoke pro-inflammatory cytokine production. These processes ultimately result in the degradation of periodontal tissues and subsequent alveolar bone resorption (ABR) (Chen J. et al., 2025; Zhang et al., 2025; Li et al., 2026a).

Among these signaling mechanisms, NF-κB functions as a pivotal mediator of inflammatory responses. Activation of NF-κB by P. gingivalis-derived LPS enhances the secretion of pro-inflammatory cytokines, such as tumor necrosis factor-alpha (TNF-α), interleukin-1 beta (IL-1β), and interleukin-6 (IL-6), further amplifying inflammation and aggravating tissue injury (Mao et al., 2016; Yang S.-Y. et al., 2024). Additionally, the activation of inflammasomes, particularly NLRP3, significantly intensifies inflammation. Activated NLRP3 inflammasomes elevate caspase-1 activity, facilitating the maturation and secretion of IL-1β and IL-18, which are closely associated with ABR (Liu et al., 2024c; Xiu et al., 2025).

Beyond classical inflammatory signaling, oxidative stress also critically contributes to periodontitis progression. During bacterial clearance, inflammatory cells produce excessive ROS, directly damaging periodontal tissues and activating inflammation-related pathways, including NF-κB, creating a positive feedback loop (Bai et al., 2024; Chen et al., 2024; Zhu et al., 2025). Collectively, these inflammatory and oxidative mechanisms reshape the local periodontal microenvironment and influence immune cell function.

Macrophage polarization represents another essential factor regulating inflammation and tissue repair. Macrophages polarize into pro-inflammatory (M1) or anti-inflammatory/pro-regenerative (M2) phenotypes under different stimuli (Peng S. et al., 2024). During the initial phase of periodontitis, pro-inflammatory M1 macrophages predominate, supporting immune defense through cytokine secretion. However, sustained activation of M1 macrophages exacerbates tissue damage (Peng S. et al., 2024; Ming et al., 2025; Li et al., 2026a; Wang X. et al., 2026). In contrast, anti-inflammatory M2 macrophages facilitate the resolution of inflammation and tissue repair by releasing growth factors and anti-inflammatory cytokines (Peng S. et al., 2024; Chen J. et al., 2025).

### 2.2 Imbalance of bone metabolism and alveolar bone resorption (ABR)

The hallmark feature of periodontitis, progressive alveolar bone destruction, results primarily from disrupted equilibrium between excessive bone resorption mediated by osteoclasts and insufficient bone formation mediated by osteoblasts (Hienz et al., 2015). Osteoclast differentiation and functional activity are tightly controlled by numerous signaling pathways, notably the receptor activator of nuclear factor-κB ligand (RANKL), its receptor RANK, and the osteoprotegerin (OPG) regulatory axis (Hienz et al., 2015; Alghamdi et al., 2023). RANKL, predominantly secreted by activated T cells, osteoblasts, and periodontal ligament cells, interacts with RANK receptors expressed on osteoclast precursors and mature osteoclasts, promoting their differentiation and activation (Hienz et al., 2015; Alghamdi et al., 2023). Conversely, OPG functions as a soluble decoy receptor that binds competitively to RANKL, thereby inhibiting the RANKL-RANK interaction and suppressing osteoclastogenesis (Zhao et al., 2024). Under normal physiological conditions, balanced RANKL and OPG expression maintains bone homeostasis (Hienz et al., 2015).

This equilibrium is disturbed in the inflammatory periodontal environment, where pro-inflammatory cytokines, such as TNF-α, IL-1β, and IL-6, along with bacterial LPS, enhance RANKL expression and suppress OPG production. This imbalance increases the RANKL/OPG ratio, accelerating osteoclast formation and subsequent alveolar bone destruction (Hienz et al., 2015; Alghamdi et al., 2023; Zhao et al., 2024). Osteoclast-mediated bone resorption relies on critical proteolytic enzymes, notably cathepsin K (CTSK), which is involved in collagen matrix degradation and directly correlates with bone-resorptive activities (Zhou et al., 2024b).

**FIGURE 1:**
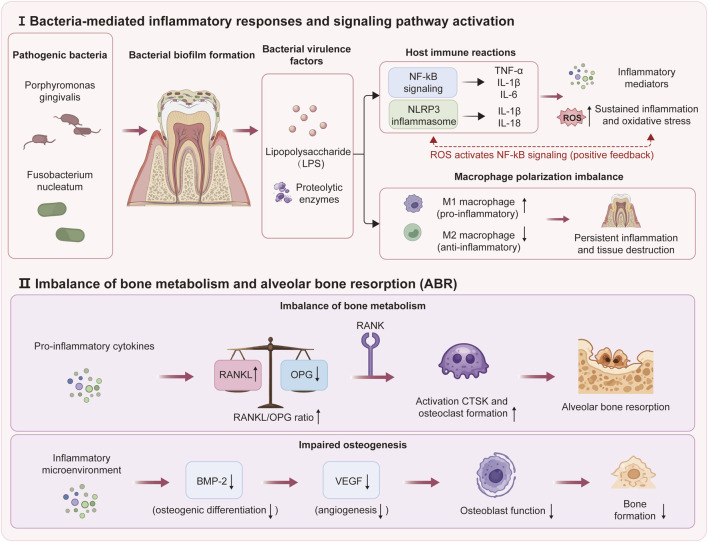
The pathogenesis of periodontitis.

Additionally, impaired osteogenesis exacerbates the imbalance of bone metabolism observed in periodontitis. Under inflammatory conditions, osteogenic differentiation is often compromised, intensifying bone loss. Bone morphogenetic protein-2 (BMP-2), essential for osteogenic differentiation of mesenchymal stem cells, shows reduced osteogenic efficacy in inflammatory environments (Huang et al., 2014). Moreover, vascular endothelial growth factor (VEGF), secreted by osteoblasts, plays a crucial role in coupling angiogenesis with osteogenesis. Dysregulated VEGF expression adversely affects periodontal regeneration (Hu and Olsen, 2016; Yang S. et al., 2024). Consequently, enhanced osteoclastic activity combined with impaired osteoblast function contributes significantly to the progressive loss of alveolar bone during periodontitis (Figure 1).

## 3 Design requirements and therapeutic advantages of hydrogels in periodontitis treatment

Current therapeutic approaches for periodontitis mainly focus on controlling dental plaque biofilms and suppressing inflammation. Non-surgical periodontal therapy (NSPT), especially supragingival scaling and subgingival scaling and root SRP, is the cornerstone of treatment, as it mechanically removes plaque and calculus to reduce bacterial load (Joseph et al., 2023; Bechir et al., 2025). Antimicrobial agents, such as antibiotics, are commonly employed adjunctively to enhance antibacterial efficacy. However, their overall clinical benefits and potential risks, including antimicrobial resistance, remain controversial (Botelho et al., 2025; Zubair, 2025). Emerging adjunctive treatments, including probiotics and laser-assisted periodontal therapy, have also been explored to improve clinical outcomes further (Baddouri and Hannig, 2024; Bian et al., 2025).

**FIGURE 2:**
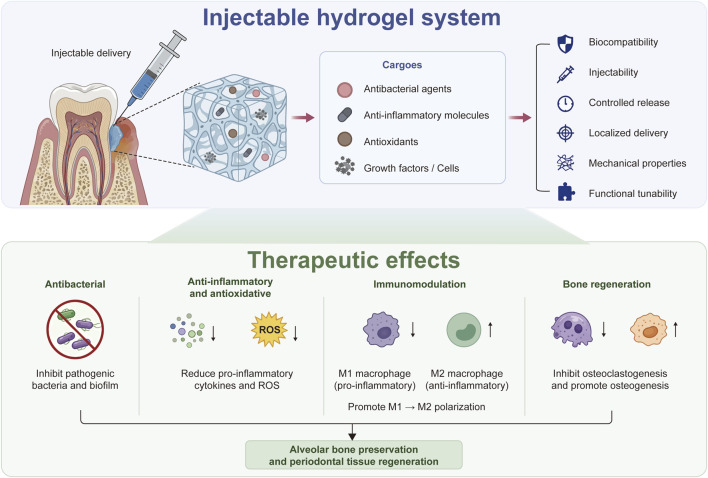
The hydrogel-based therapeutic strategies of periodontitis.

Despite these advancements, existing therapies have limitations within the complex periodontal microenvironment, particularly regarding the short retention time of locally delivered drugs and inadequate regenerative capacity (Nakajima et al., 2025; Pan Q. et al., 2025). Therefore, biomaterials designed for periodontitis treatment must meet several key criteria. First, excellent biocompatibility is necessary to prevent adverse immune responses or cytotoxicity upon contact with periodontal tissues (El-Nablaway et al., 2024; Dal-Fabbro et al., 2025). Second, injectability permits minimally invasive administration into narrow and irregular periodontal pockets, enabling stable three-dimensional structure formation for effective defect filling and local retention (Shirbhate and Bajaj, 2022; Tan et al., 2023). Additionally, functional tunability facilitates incorporation of bioactive agents, such as antibacterial drugs, anti-inflammatory molecules, growth factors, or cells, thereby achieving multifunctional therapeutic outcomes, including antibacterial activity, immunomodulation, and tissue regeneration (Li L. et al., 2025). Furthermore, appropriate mechanical properties provide structural support under complex oral conditions, and controllable biodegradability ensures gradual degradation aligned with tissue repair processes (Xu S. et al., 2023; Hu et al., 2024; Pan P. et al., 2025). Given these requirements, increasing attention has been directed toward highly customizable biomaterials, among which injectable hydrogels have emerged as promising candidates for localized therapy and periodontal regeneration (El-Nablaway et al., 2024; Dal-Fabbro et al., 2025) (Figure 2).

Compared with traditional periodontal materials such as bone grafts, which typically have limited functionality, hydrogels offer versatile platforms for multifunctional therapy by integrating diverse bioactive molecules or nanomaterials. Consequently, hydrogels can simultaneously provide antibacterial, anti-inflammatory, antioxidative, immunomodulatory, and regenerative effects, enabling comprehensive intervention in the pathological processes of periodontitis (Xu Y. et al., 2023; Luo et al., 2024; Ge et al., 2025a). Additionally, hydrogels can mimic the ECM, supplying a three-dimensional microenvironment for periodontal ligament stem cells and promoting the formation of new bone, cementum, and functional periodontal ligament (Chien et al., 2018; Ivanov et al., 2023).

Additionally, traditional biomaterials typically necessitate surgical implantation and lack flexibility to conform precisely to the irregular anatomy of periodontal defects (Harsas et al., 2025). In contrast, injectable hydrogel formulations provide a minimally invasive method to effectively fill complex periodontal pockets and irregular bone cavities, significantly enhancing adaptability and minimizing surgical trauma (Ran et al., 2024). Moreover, conventional local antibiotic treatments often result in rapid drug release, failing to sustain therapeutic concentrations. Systemically administered antibiotics, however, carry risks of systemic side effects and can contribute to antimicrobial resistance development (Zhou et al., 2026). Injectable hydrogels, however, enable sustained and controlled release of therapeutic agents, offering more precise localized treatment through responsiveness to changes in the periodontal microenvironment (Quan et al., 2025).

Injectable hydrogels are increasingly being developed as multifunctional therapeutic platforms rather than simple drug delivery carriers. Beyond antibacterial activity, current designs seek to incorporate antioxidative, immunomodulatory, and regenerative functions within a single system. An EGCG-Cu-loaded injectable hydrogel was reported to suppress Porphyromonas gingivalis, reduce excessive reactive oxygen species, promote macrophage polarization toward the M2 phenotype, and enhance osteogenic differentiation and periodontal tissue regeneration. Such multifunctional systems highlight the potential of simultaneously targeting microbial infection, immune dysregulation, oxidative stress, and tissue destruction within the periodontal microenvironment. The integration of these therapeutic functions may represent an important direction for the development of next-generation hydrogels for periodontitis treatment (Hu et al., 2025).

## 4 Classification of injectable hydrogels in periodontitis therapy

**FIGURE 3:**
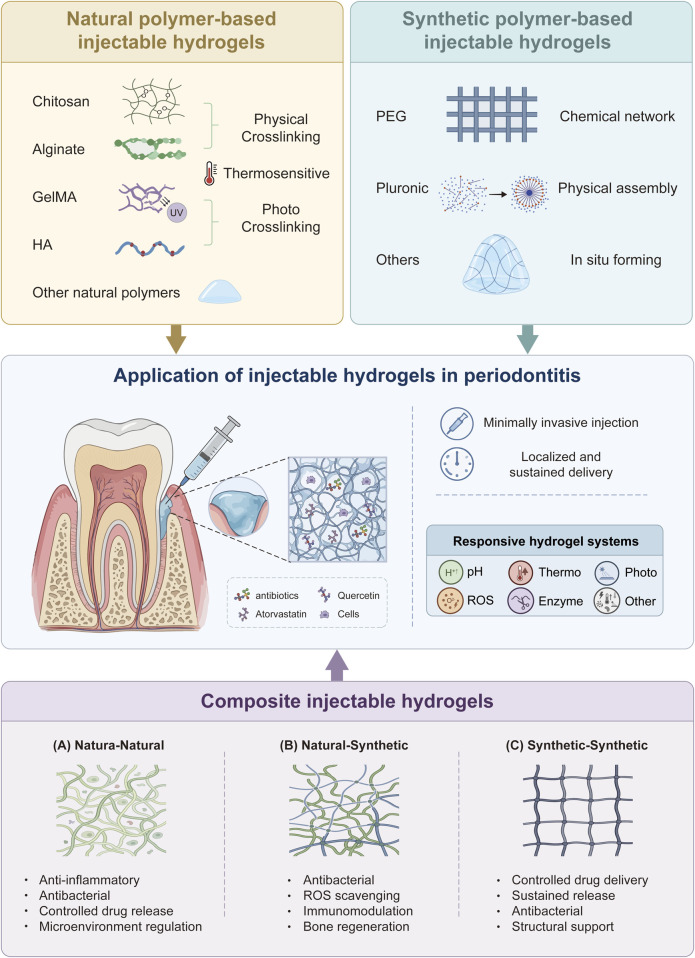
Injectable hydrogels for periodontitis: classification and applications.

Injectable hydrogels are commonly delivered as precursor solutions and may undergo *in situ* gelation after injection, allowing minimally invasive administration into periodontal defects (Pan Q. et al., 2025). This mode of delivery is well suited for periodontitis treatment because periodontal pockets and intrabony defects usually have irregular shapes that are difficult to manage using conventional preformed biomaterials. After injection, hydrogels can better adapt to the defect area, which helps improve local retention and maintain therapeutic effects at the lesion site (Yin et al., 2025). Depending on the material system, gelation may occur through thermosensitive transition, ionic crosslinking, dynamic covalent bonding, or photo-induced crosslinking (Ran et al., 2024; Pan Q. et al., 2025). Meanwhile, from the perspective of material composition, these hydrogels are generally divided into natural polymer-based, synthetic polymer-based, and composite polymer-based hydrogels (Dal-Fabbro et al., 2025; Wang S. et al., 2026) (Figure 3).

### 4.1 Natural polymer-based injectable hydrogels

Natural polymer-based injectable hydrogels have been widely investigated in periodontitis therapy because of their favorable biocompatibility, biodegradability, and ECM-mimicking properties (El-Nablaway et al., 2024). Common natural polymers, including chitosan, alginate, gelatin, and hyaluronic acid, can support localized drug delivery and periodontal tissue repair. However, their relatively weak mechanical stability and limited long-term durability often require further modification or combination with other functional materials.

#### 4.1.1 Chitosan-based injectable hydrogels

Chitosan is a natural cationic polysaccharide derived from the deacetylation of chitin. It has been extensively applied in periodontitis treatment owing to its favorable biocompatibility, biodegradability, antibacterial activity, and wound-healing capabilities (Xu S. et al., 2023; Li et al., 2025d; Wang H. et al., 2025; Yu X. et al., 2026). Chitosan is a natural cationic polysaccharide that has attracted considerable attention in injectable hydrogel systems owing to its favorable biocompatibility, biodegradability, and intrinsic antibacterial properties (Li et al., 2025d). One notable advantage of chitosan-based hydrogels lies in their capacity to undergo mild gelation through physical crosslinking mechanisms, such as temperature-responsive sol-gel transitions, eliminating the need for chemical crosslinkers (Li et al., 2025d; Wang H. et al., 2025). Previous research has indicated that thermoresponsive hydrogels composed of chitosan and β-glycerophosphate remain fluid at ambient temperature yet rapidly transition to a gel state at body temperature (Wang H. et al., 2025). This property enables minimally invasive injection into periodontal pockets, facilitating *in situ* gel formation that closely adapts to irregular defect sites for effective local retention. Additionally, chitosan-based hydrogels serve as multifunctional delivery platforms for antibacterial agents, anti-inflammatory molecules, or bioactive factors. These systems inhibit bacterial growth, modulate inflammation, and promote cellular activities, contributing to inflammation control, oxidative stress regulation, and tissue repair (Xu S. et al., 2023; Cai et al., 2024; Li et al., 2025d; 2026b; Wang H. et al., 2025; Yu X. et al., 2026).

#### 4.1.2 Alginate-based injectable hydrogels

Sodium alginate, an anionic polysaccharide derived from brown seaweed, is distinguished by its rapid gelation ability via ionic interactions with divalent cations (e.g., Ca^2+^). This characteristic enables alginate hydrogels to form under gentle conditions, making them highly suitable for localized injectable treatments in periodontitis, easily conforming to the irregular contours of periodontal pockets and efficiently delivering therapeutic agents (Bashir et al., 2024; Fang et al., 2024; Li C. et al., 2025; Wang J. et al., 2025). Moreover, ionically crosslinked alginate hydrogels form without organic solvents or chemical crosslinking agents, resulting in favorable biocompatibility (Bashir et al., 2024). Consequently, alginate hydrogels effectively serve as local drug delivery systems. Alginate-based injectable hydrogels loaded with antimicrobial agents undergo *in situ* gelation within periodontal pockets, maintaining sustained drug release and effective antibacterial concentrations (Zussman and Zilberman, 2022). Additionally, incorporating functional components significantly enhances therapeutic efficacy. Alginate hydrogels integrated with silver-based metal-organic frameworks exhibit antibacterial properties through Ag^+^ release and ROS generation, disrupting bacterial membrane integrity, interfering with intracellular metabolism, and inhibiting biofilm formation. Simultaneously, these systems promote endothelial cell migration and angiogenesis and reduce inflammatory cytokine levels *in vivo*, thereby alleviating ABR through synergistic antibacterial and regenerative effects (Wang J. et al., 2025).

#### 4.1.3 Gelatin/gelatin methacryloyl (GelMA)-based injectable hydrogels

Gelatin, a natural protein derived from collagen hydrolysis, is extensively used in biomedical fields owing to its superior biocompatibility, biodegradability, and cell adhesion capabilities (Yue et al., 2015). Nevertheless, pure gelatin-based hydrogels demonstrate limited mechanical stability and are prone to rapid degradation in physiological environments. Therefore, gelatin is typically chemically modified by introducing photocrosslinkable methacrylate groups, producing gelatin methacryloyl (GelMA). GelMA maintains gelatin’s biological activity while enabling photo-induced crosslinking, resulting in hydrogels with adjustable mechanical strength and structural stability (Yue et al., 2015; Hou et al., 2025). Additionally, GelMA preserves arginine-glycine-aspartic acid (RGD) motifs, promoting interactions with periodontal tissues, particularly periodontal ligament cells, enhancing cell adhesion, proliferation, and differentiation. The photocrosslinking process precisely controls gelation timing, and the methacrylation degree enables tunable degradation (Hou et al., 2025).

GelMA has been applied in various forms for periodontitis treatment, including stem cell and drug delivery systems and multifunctional composite hydrogels. GelMA hydrogels loaded with hypoxia-preconditioned stem cells from human exfoliated deciduous teeth (SHEDs) exhibit favorable biocompatibility and osteogenic differentiation capability, thus promoting periodontal regeneration (Chen Y. et al., 2025). Furthermore, GelMA-based systems can achieve multifunctional therapeutic effects. GelMA hydrogels containing antimicrobial peptides and CeO_2_ nanoparticles effectively scavenge ROS and regulate macrophage polarization, promoting alveolar bone regeneration (Luo et al., 2025). Although GelMA hydrogels demonstrate promising therapeutic potential in preclinical studies, their relatively low mechanical strength limits standalone applications in the complex oral environment. Thus, they are often combined with other materials to improve structural stability and functional performance (Liu et al., 2022; Hou et al., 2025).

#### 4.1.4 Hyaluronic acid-based injectable hydrogels

Hyaluronic acid (HA), an endogenous polysaccharide component of the ECM, is widely adopted in hydrogel formulations for periodontal therapy due to its inherent biocompatibility, biodegradability, anti-inflammatory effects, and ability to facilitate wound healing (Hu et al., 2024; Zhu et al., 2025). However, native HA exhibits limited stability and mechanical strength. Thus, chemical modifications, such as methacrylation, introduce photocrosslinkable groups, yielding methacrylated hyaluronic acid (HAMA) with enhanced structural stability (Hu et al., 2024). Furthermore, incorporating dynamic chemical bonds or combining HA with thermosensitive materials creates functional hydrogels responsive to ROS or temperature. These properties enable better adaptation to the periodontal microenvironment and controlled drug release (Liu et al., 2024b; Zhu et al., 2025).

Consequently, HA-based functional hydrogels have become multifunctional therapeutic platforms. Incorporating nanoparticles into a Pluronic F127/hyaluronic acid methacrylate (HAMA) double-network hydrogel achieves simultaneous antibacterial effects, ROS scavenging, and inflammation modulation, thus alleviating ABR (Liu et al., 2024b). Additionally, hydrogels with dynamic networks incorporating antibacterial and antioxidant components modulate macrophage polarization and improve the inflammatory microenvironment (Ge et al., 2025a; Zhu et al., 2025). Furthermore, combining HA with osteogenic components like bioactive glass promotes osteogenic gene expression while providing antibacterial and anti-inflammatory benefits, enhancing periodontal regeneration (Hu et al., 2024).

#### 4.1.5 Other natural polymer-based injectable hydrogels

In addition to chitosan, alginate, gelatin, and hyaluronic acid, pectin has also been explored as a natural polymer for periodontal therapy. It is a biodegradable polysaccharide that can form gels in the presence of calcium ions, making it suitable for *in situ* hydrogel preparation. Studies have shown that pectin-based hydrogels have promising drug delivery capabilities and may help reduce periodontal inflammation, supporting their use in localized therap (Mallamma et al., 2024). Nevertheless, current studies regarding pectin-based hydrogels for periodontal disease are relatively scarce and primarily exploratory.

### 4.2 Synthetic polymer-based injectable hydrogels

Synthetic polymer-based injectable hydrogels are widely used in periodontitis therapy because their mechanical properties, degradation behavior, and drug release profiles can be more easily regulated. Common synthetic materials, including PEG-, PLGA-, and Pluronic-based systems, are mainly applied as injectable drug delivery platforms and functional carriers. However, since most synthetic polymers lack intrinsic biological activity, they are often combined with bioactive molecules or other functional materials to improve therapeutic performance.

#### 4.2.1 Poly (ethylene glycol) (PEG)-based injectable hydrogels

Poly (ethylene glycol) (PEG) is a synthetic polymer widely utilized in biomedical applications. PEG forms hydrogels through click chemistry or photocrosslinking reactions, enabling tunable mechanical properties and degradation rates. These properties render PEG suitable for injectable applications, facilitating adaptation to irregular periodontal pockets and effective local drug delivery (Elgendy et al., 2023; Huang et al., 2023; Xia et al., 2025). However, PEG hydrogels inherently lack antibacterial and regenerative bioactivity. Owing to their excellent biocompatibility and low immunogenicity, PEG hydrogels commonly serve as drug carriers or tissue engineering scaffolds (Elgendy et al., 2023; Xia et al., 2025). To overcome this limitation, composite strategies frequently incorporate antibacterial, anti-inflammatory, and osteogenic functionalities. A thermosensitive poly (lactide-co-glycolide acid) and polyethylene glycol triblock copolymer hydrogel loaded with TRAF-STOP effectively inhibits osteoclast differentiation by suppressing the CD40L-CD40-TRAF6 axis and NF-κB pathway (Huang et al., 2023). Furthermore, PEG-based hydrogels exhibit enhanced therapeutic outcomes, including antibacterial properties and promotion of bone regeneration, by incorporating bioactive drugs or nanoparticles. A eugenol-loaded PEGylated cubosome hydrogel containing atorvastatin significantly improved clinical outcomes, reducing periodontal probing depth and bleeding indices in periodontitis patients (Elgendy et al., 2023). Despite their superior mechanical properties, customizable physicochemical characteristics, and effective drug encapsulation abilities, PEG hydrogels typically necessitate functional modifications or composite designs to achieve multifunctional therapeutic objectives, such as simultaneous antimicrobial, anti-inflammatory, and regenerative capabilities (Elgendy et al., 2023; Xia et al., 2025).

#### 4.2.2 Poloxamer (Pluronic)-based injectable hydrogels

Poloxamers, commonly termed Pluronics, are triblock amphiphilic copolymers consisting of poly (propylene oxide) and poly (ethylene oxide) segments. They exhibit thermoresponsive gelation characteristics, existing as liquids at lower temperatures and swiftly converting to gel form at physiological temperatures, facilitating their injectable *in situ* gelation properties (El-Nablaway et al., 2024; Ge et al., 2025b; Shin et al., 2025; Wang G. et al., 2025; Yu X. et al., 2026). As non-ionic copolymers, Pluronics undergo gelation without requiring chemical crosslinking agents, thereby retaining favorable biocompatibility and suitable mechanical integrity (El-Nablaway et al., 2024; Yu X. et al., 2026).

Due to these characteristics, Pluronic-based hydrogels primarily function as drug delivery platforms for periodontitis, enabling antibacterial, anti-inflammatory, and regenerative effects via incorporated bioactive agents. Advanced functional strategies, such as stimulus-responsive release, targeted delivery, and multifunctional integration, further enhance their therapeutic efficacy (Pan Q. et al., 2025). A quercetin-loaded ROS-responsive hollow mesoporous silica nanoparticle system was combined with 4-terpineol-modified thermosensitive Pluronic F127 to form a composite hydrogel. This system effectively scavenged ROS in the inflammatory microenvironment, alleviated endoplasmic reticulum stress in periodontal ligament stem cells, and reduced apoptosis, thereby indirectly enhancing their osteogenic differentiation potential (Wang G. et al., 2025). Similarly, a simvastatin-loaded pyrophosphorylated Pluronic F127 thermosensitive hydrogel exhibits enhanced bone affinity and local drug retention, effectively inhibiting ABR (Chen et al., 2021). Additionally, a chitosan-grafted Pluronic F127-based nanocomposite hydrogel loaded with doxycycline demonstrates antibacterial activity, promotes macrophage M2 polarization, scavenges ROS, and enhances osteogenesis, ultimately improving alveolar bone regeneration (Yu X. et al., 2026). Overall, Pluronic-based hydrogels enable multilevel functional modulation of the periodontal microenvironment and show promising potential for periodontitis treatment.

#### 4.2.3 Other synthetic polymer-based injectable hydrogels

Other synthetic polymer-based hydrogels have attracted attention for bone defect repair because of their favorable mechanical properties and controllable degradation behavior. Injectable in situ-forming PLGA hydrogels can transform into porous scaffolds after administration, providing mechanical support at defect sites. In addition, the incorporation of magnesium particles helps neutralize the acidic degradation products of PLGA, thereby improving the local microenvironment (Zhou et al., 2024a). Additionally, carboxymethyl cellulose-based hydrogels are promising candidates due to excellent biocompatibility and functional versatility. An injectable cationic hydrogel constructed by grafting cationic poly(amidoamine) dendrimers onto oxidized carboxymethyl cellulose can capture bacterial LPS and extracellular DNA, inhibiting the TLR4/9-NF-κB pathway, alleviating inflammation, and ultimately reducing ABR (Chen X. et al., 2025).

### 4.3 Composite injectable hydrogels

Composite injectable hydrogels have emerged as an important strategy for periodontal therapy by integrating hydrogel matrices with secondary functional components, including nanoparticles, microspheres, antibiotics, and photothermal agents. Compared with single-component systems, these composite platforms exhibit improved structural adaptability, local retention, and functional integration within the complex periodontal microenvironment. In addition to enhancing material stability at the defect site, composite systems can simultaneously regulate bacterial infection, inflammatory responses, and tissue regeneration. Therefore, compared with the single-component systems discussed above, composite injectable hydrogels are increasingly designed to integrate multiple therapeutic functions within a single platform to achieve synergistic regulation of the periodontal microenvironment.

#### 4.3.1 Natural-natural polymer composite injectable hydrogels

Natural polymer-based composite injectable hydrogels enable coordinated regulation of infection, inflammation, oxidative stress, and tissue regeneration within the periodontal microenvironment. Enzyme-responsive GelMA systems incorporating minocycline-loaded chitosan nanoparticles further illustrate how natural polymer combinations can prolong local antibacterial delivery and support periodontal bone defect repair (You et al., 2023). A MCC950-loaded GelMA/ε-polylysine double-crosslinked hydrogel suppressed NOD-like receptor family pyrin domain-containing 3 (NLRP3) inflammasome-mediated inflammatory cascades and promoted inflammation resolution (Liu et al., 2024c). Similarly, hydrogels co-delivering curcumin and glycyrrhizic acid enhanced anti-inflammatory activity and tissue regeneration through complementary antioxidant and immunomodulatory effects (Chen et al., 2026). An interpenetrating hydrogel network loaded with epigallocatechin gallate and a nitric oxide donor simultaneously scavenged ROS, inhibited bacterial growth, and promoted macrophage M2 polarization, thereby enhancing alveolar bone regeneration (Yu J. et al., 2026).

#### 4.3.2 Natural-synthetic polymer composite injectable hydrogels

Natural-synthetic composite injectable hydrogels combine the bioactivity of natural polymers with the structural tunability of synthetic components, allowing improved stability and local retention within periodontal lesions. A hydrogel containing two-dimensional titanium carbide MXene nanosheets and poly-L-lysine inhibited Porphyromonas gingivalis, reduced ROS accumulation, and promoted alveolar bone regeneration in a rat periodontitis model (Yu et al., 2024). Similarly, incorporation of manganese ferrite nanozymes into PVA/gelatin hydrogels enhanced antioxidative activity and promoted bone regeneration through regulation of the Zipcode-binding protein 1/β-catenin pathway (Zhang et al., 2026). Multicomponent network designs further improve functional integration within the periodontal microenvironment. A double-network hydrogel composed of alginate, four-arm PEG, and Ca^2+^-tannic acid nanocomposites exhibited antibacterial activity and osteogenic potential while alleviating inflammatory responses (Ouyang et al., 2025). Combining natural and synthetic polymer networks with nanomaterials or bioactive factors also enables responsive drug delivery and coordinated antibacterial, anti-inflammatory, and osteogenic effects during periodontal repair (Gui et al., 2025; Yin et al., 2025).

#### 4.3.3 Synthetic-synthetic polymer composite injectable hydrogels

Synthetic polymer composite systems allow precise regulation of degradation behavior and drug release kinetics, making them suitable platforms for injectable local delivery. Thermosensitive hydrogels based on PLGA-PEG-PLGA can regulate sol-gel transition and sustained drug release through adjustments in polymer composition and concentration (Khodaverdi et al., 2012). Meanwhile, PEG-PLA-based nanocarriers prolong drug residence time in gingival crevicular fluid and improve local therapeutic efficacy (Yao et al., 2014). Moreover, PLGA-PCL synthetic polymers can also be fabricated into nanoparticles or nanofibers with controlled-release and antibacterial properties, providing versatile structural components for local delivery systems (Toledano-Osorio et al., 2024).

Although fully synthetic composite injectable hydrogels remain relatively underexplored in periodontitis treatment, combining synthetic hydrogel matrices with synthetic nanocarriers or structural units may further improve local drug delivery and therapeutic regulation. Meanwhile, the comparative characteristics of different injectable hydrogel categories for periodontitis treatment have been shown in Table 1.

**TABLE 1:** Comparative characteristics of injectable hydrogels for periodontitis treatment.

Polymer type	Hydrogel category	Representative composition	Stimuli-responsiveness	Main advantages	Current limitations	Representative applications	Level of clinical readiness	Representative references
Natural polymer-based injectable hydrogels	Chitosan-based injectable hydrogels	Chitosan/β-GP and modified chitosan systems	Thermoresponsive; pH-responsive in modified chitosan systems	Good biocompatibility, injectability, and intrinsic antibacterial activity	Weak mechanical stability and relatively rapid degradation	Local drug delivery; antibacterial therapy; inflammation regulation; periodontal regeneration	Preclinical	Xu et al. (2023a), Li et al. (2025d), Wang et al. (2025b)
	Alginate-based injectable hydrogels	Alginate/Ca2+ and alginate-based composite systems	Ion-responsive	Mild gelation conditions and favorable injectability	Limited long-term stability and weak intrinsic osteogenic activity	Antibacterial therapy; inflammation regulation; alveolar bone protection	Preclinical	Fang et al. (2024), Wang et al. (2025c)
	Gelatin/GelMA-based injectable hydrogels	GelMA and nanoparticle-loaded GelMA systems	Photoresponsive	Favorable cell affinity, ECM-mimicking properties, and regenerative potential	Relatively weak mechanical properties when used alone	Stem cell delivery; ROS scavenging; periodontal tissue regeneration	Preclinical	Yue et al. (2015), Liu et al. (2022), Luo et al. (2025)
	HA/HAMA-based injectable hydrogels	HAMA and PF127/HAMA systems	Photoresponsive; ROS-responsive in composite systems	Good anti-inflammatory and wound-healing potential	Native HA exhibits poor structural stability	Microenvironment modulation; inflammation regulation; bone regeneration	Preclinical	Hu et al. (2024), Liu et al. (2024b), Zhu et al. (2025)
Synthetic polymer-based injectable hydrogels	PEG-based injectable hydrogels	PEG and PLGA-PEG-PLGA systems	Thermoresponsive; pH-responsive	Tunable mechanical properties and controllable drug release	Limited intrinsic bioactivity	Sustained local drug delivery; periodontal regeneration	Preclinical	Huang et al. (2023), Xia et al. (2025)
	Pluronic-based injectable hydrogels	Pluronic F127-based systems	Thermoresponsive; ROS-responsive in composite systems	Rapid sol-gel transition and convenient injectable delivery	Limited long-term retention and bioactivity as standalone systems	Smart local drug delivery; ROS regulation; antibacterial and osteogenic therapy	Preclinical	Chen et al. (2021), Wang et al. (2025a)
	Other synthetic polymer-based injectable hydrogels	PLGA- and PAMAM-based systems	Degradation-responsive; adsorption-based immune regulation	Good structural support and controllable degradation behavior	Acidic degradation products and limited regenerative bioactivity	Bone defect repair; inflammatory regulation; localized drug delivery	Preclinical	Zhou et al. (2024a), Chen et al. (2025b)
Composite polymer-based injectable hydrogels	Natural-natural composite injectable hydrogels	GelMA/chitosan nanoparticle systems; GeLMA/ε-polylysine hydrogels; EGCG/NO-loaded interpenetrating hydrogel networks	Enzyme-responsive; ROS-responsive	Excellent biocompatibility and synergistic bioactivity; enhanced antibacterial, anti-inflammatory, and regenerative functions	Limited mechanical strength and batch-to-batch variability	Antibacterial therapy; inflammation regulation; periodontal regeneration	Preclinical	You et al. (2023), Liu et al. (2024c), Yu et al. (2026a)
	Natural-synthetic composite injectable hydrogels	MXene/PLL hydrogels; PVA/gelatin-manganese ferrite nanozyme hydrogels; alginate/4-arm PEG/Ca2+ tannic acid nanocomposite systems	ROS-responsive; antioxidant-responsive	Combines bioactivity and structural tunability; improved stability and local retention	Increased formulation complexity and potential nanomaterial-related biosafety concerns	Antibacterial therapy; ROS regulation; osteogenesis; periodontal regeneration	Preclinical	Yu et al. (2024), Zhang et al. (2026), Ouyang et al. (2025)
	Synthetic-synthetic composite injectable hydrogels	PLGA-PEG-PLGA systems; PEG-PLA nanocarriers; PLGA-PCL systems	Thermoresponsive; controlled-release	Precisely tunable degradation behavior and drug release kinetics	Limited intrinsic bioactivity and reliance on functional modification	Sustained local drug delivery; controlled-release therapy	Preclinical	Khodaverdi et al. (2012), Toledano-Osorio et al. (2024)

## 5 Stimuli-responsive drug delivery properties of injectable hydrogels

Stimuli-responsive injectable hydrogels have attracted attention in periodontitis therapy because the periodontal microenvironment undergoes dynamic pathological changes during disease progression, including alterations in pH, oxidative stress, enzyme activity, and inflammatory conditions. Compared with conventional sustained-release systems, these hydrogels enable localized drug release in response to specific microenvironmental stimuli. In addition to improving local retention at periodontal defects, stimuli-responsive systems can further regulate antibacterial, anti-inflammatory, and regenerative processes within the periodontal microenvironment. These pathological alterations provide the biological basis for the design of stimuli-responsive injectable hydrogels (Figure 3).

### 5.1 pH-responsive drug delivery of injectable hydrogels

Periodontitis is associated with dynamic alterations in the local pH microenvironment. Under healthy conditions, gingival crevicular fluid (GCF) remains close to neutral, whereas disease progression is often accompanied by elevated GCF pH (Xia et al., 2025). Localized acidic regions may also arise within infected lesions owing to bacterial metabolism and inflammatory activity (Cai et al., 2024; Li et al., 2025d), indicating substantial spatial and temporal heterogeneity within periodontal pockets. Injectable pH-responsive hydrogels have therefore been explored for localized drug delivery in response to these pathological fluctuations. Dynamic Schiff-base-crosslinked Carboxymethyl Chitosan-Oxidized Dextran hydrogels enabled pH-responsive drug release together with self-healing behavior, allowing adaptation to the changing periodontal microenvironment (Cai et al., 2024). Incorporation of liraglutide further endowed the pH-responsive hydrogel system with sustained local delivery and ROS-scavenging capability under diabetic periodontal conditions (Li et al., 2025d). In parallel, alkaline-responsive PEG-based hydrogels were designed to match the elevated pH of diseased GCF, enabling pH-triggered rutin release and suppression of inflammatory responses (Xia et al., 2025). Recently, incorporation of metal-organic frameworks into dual-crosslinked injectable hydrogels extended pH-responsive systems toward multi-stimuli-regulated drug delivery within periodontal defects (Luo et al., 2024).

### 5.2 ROS-responsive drug delivery of injectable hydrogels

Excessive ROS accumulation contributes to persistent inflammation, mitochondrial dysfunction, and impaired tissue repair within chronic inflammatory microenvironments, highlighting oxidative stress as an important therapeutic target (Yu et al., 2025). ROS-responsive injectable hydrogels have therefore been developed either to trigger drug release under oxidative conditions or to directly scavenge excessive ROS within periodontal lesions. Systems incorporating ROS-cleavable linkages, particularly aryl boronate ester bonds, enable localized release of therapeutic agents in response to oxidative stress (Li J. et al., 2024; 2025c). A chitosan-based injectable hydrogel loaded with caffeic acid phenethyl ester through boronate ester linkages achieved ROS-responsive drug release while suppressing NF-κB-mediated inflammatory signaling and activating the Wnt/β-catenin pathway, thereby promoting osteogenic differentiation within periodontal defects (Peng C. et al., 2024). Integration of ROS-responsive nanocarriers into thermosensitive injectable hydrogels further improved spatiotemporal control over local drug release under oxidative conditions (Wang G. et al., 2025). Some injectable hydrogels instead regulate oxidative stress through antioxidant components rather than ROS-cleavable structures. A GelMA/oxidized hyaluronic acid double-network injectable hydrogel loaded with grape seed extract-zinc nanoparticles effectively scavenged mitochondrial ROS, restored redox homeostasis, and regulated macrophage polarization, ultimately alleviating inflammatory responses and promoting periodontal tissue regeneration (Su et al., 2026).

### 5.3 Thermoresponsive drug delivery of injectable hydrogels

Thermosensitive injectable hydrogels remain fluid during administration and rapidly undergo sol-gel transition at physiological temperature, which improves retention within periodontal pockets and prolongs local drug release. Chitosan/β-GP systems are among the most widely investigated thermoresponsive platforms for periodontal therapy because gelation can occur rapidly after injection through temperature-dependent ionic interactions. Incorporation of gelatin further shortens gelation time and enhances hydrogel stability. A mesalazine-loaded chitosan/β-GP/gelatin hydrogel reduced inflammatory responses and inhibited alveolar bone resorption in experimental periodontitis (Wang H. et al., 2025). Modification of the chitosan/β-GP system with sodium bicarbonate also decreased the amount of β-GP required for gel formation while maintaining rapid thermoresponsive behavior. Sustained local release of the CCL2 inhibitor bindarit subsequently suppressed pro-inflammatory cytokine expression within periodontal tissues (Jiang et al., 2026).

### 5.4 Enzyme-responsive drug delivery of injectable hydrogels

At periodontitis sites, bacterial proteases, particularly arginine-specific gingipain A secreted by Porphyromonas gingivalis, are significantly elevated and can be used as triggers for enzyme-responsive localized drug delivery (Li et al., 2025c). Enzyme-responsive injectable hydrogels are therefore commonly designed by incorporating protease-cleavable peptide sequences into hydrogel networks. In a gingipain-responsive thermosensitive hydrogel system, cleavage of peptide linkers by porphyromonas gingivalis triggered local release of antimicrobial peptides, thereby suppressing bacterial growth within periodontal pockets. The same platform also enabled sustained delivery of stromal cell-derived factor-1, which promoted recruitment and osteogenic differentiation of periodontal ligament stem cells during periodontal regeneration (Liu et al., 2021).

### 5.5 Light-responsive drug delivery of injectable hydrogels

Light-responsive injectable hydrogels have been explored for both *in situ* photocrosslinking and externally regulated drug release within periodontal defects. Photopolymerizable systems containing photoinitiators and photocrosslinkable groups enable rapid gelation after local injection (Liu et al., 2022). Composite injectable hydrogels incorporating methacrylated hyaluronic acid and mesoporous bioactive glass nanoparticles further improved hydrogel stability while supporting antibacterial and osteogenic activities (Hu et al., 2024). In addition, integration of photothermal agents into injectable hydrogel systems enabled near-infrared-responsive antibacterial therapy. A GelMA-based hybrid hydrogel containing Au nanobipyramids and mesoporous silica nanoparticles achieved sustained antibiotic release together with photothermal antibacterial effects under 808 nm irradiation, thereby improving bacterial control within periodontal pockets (Lin et al., 2020).

### 5.6 Other stimuli-responsive systems of injectable hydrogels

Beyond chemical stimuli, some injectable hydrogels have been designed to respond to mechanical cues within periodontal tissues. Piezoelectric hydrogels are of particular interest for periodontal applications because mechanical stress generated during mastication can be converted into localized electrical stimulation. A piezoelectric injectable hydrogel composed of tetragonal barium titanate nanoparticles and tilapia-derived GelMA generated piezopotentials under mechanical stimulation, leading to enhanced mitochondrial bioenergetics and ATP production in inflammatory periodontal ligament stem cells (Liu X. et al., 2024). The resulting bioelectrical stimulation promoted osteogenic differentiation and shifted macrophage polarization toward a pro-regenerative phenotype. Combined with the intrinsic anti-inflammatory activity of tilapia gelatin, the hydrogel further improved periodontal tissue regeneration in inflammatory defects (Liu X. et al., 2024).

## 6 Clinical evidence and translational progress of hydrogel-based therapies

Despite growing research interest in injectable hydrogels and local drug delivery systems for periodontitis, clinical evidence remains limited. Existing studies mainly investigate gel-based local delivery systems used adjunctively with scaling and root planing (SRP). Current clinical findings suggest that local drug delivery enhances inflammatory control and periodontal clinical outcomes. Green tea catechin gels have demonstrated significant reductions in gingival index, probing pocket depth, and clinical attachment loss compared with SRP alone (Chava and Vedula, 2013; Rattanasuwan et al., 2016). Similarly, local curcumin gel application significantly improved probing depth, plaque index, and clinical attachment levels in short-term follow-ups (Ravishankar et al., 2017). Clinical evidence also supports local antimicrobial gels; doxycycline and chlorhexidine gels combined with SRP significantly reduce probing depth and enhance clinical attachment compared with SRP alone (Verma et al., 2008). Thus, gel-based local drug delivery systems effectively complement SRP in periodontitis management, primarily controlling inflammation and improving clinical parameters. However, direct clinical evidence supporting periodontal tissue regeneration is limited.

Recent clinical trial registrations indicate ongoing interest in hydrogel-based regenerative therapies, exploring functional molecule-loaded hydrogels combined with minimally invasive surgery for intrabony defect repair or evaluating their efficacy in periodontitis and peri-implantitis treatment (NCT05653245; NCT06740123). Most current clinical studies, however, continue to focus on conventional gel-based delivery systems, including antimicrobial gels (e.g., metronidazole and nitazoxanide), natural bioactive gels (e.g., proanthocyanidins), and hyaluronic acid (NCT04983849; NCT04768530; NCT05015387; NCT03754010). Despite clinical applications of gel-based drug delivery strategies, existing studies are often limited by small sample sizes and short follow-up periods. Clinical evidence specifically addressing injectable and multifunctional hydrogel systems remains scarce, and long-term efficacy and safety require further evaluation, underscoring a persistent gap between material development and clinical translation.

## 7 Current challenges of injectable hydrogels in periodontitis treatment

### 7.1 Insufficient mechanical properties

Although injectable hydrogels show promise for localized periodontitis treatment, their inadequate mechanical strength remains a significant barrier for clinical application. Many hydrogels derived from natural polymers, such as gelatin and ECM components, rely on weak interactions for structural integrity. Consequently, they are unable to withstand prolonged mechanical stress in the oral environment (Gui et al., 2025; Ma S. et al., 2025; Peng et al., 2026). Additionally, the moist, dynamic oral environment leads to structural instability and accelerated degradation, compromising their long-term effectiveness (Gui et al., 2025). Materials placed in periodontal pockets must also withstand mechanical loading and salivary flow, further exacerbating this challenge (Gui et al., 2025).

### 7.2 Limited functionality

Periodontitis pathogenesis involves bacterial infection, dysregulated inflammation, and bone metabolism imbalance (Hajishengallis et al., 2020). Ideal therapeutic materials should thus simultaneously exhibit antibacterial, anti-inflammatory, and regenerative functions. However, most injectable hydrogels provide only a single therapeutic function, such as antibacterial or osteogenic activity alone (Xu S. et al., 2023). Although recent studies emphasize multifunctional biomaterials to modulate complex inflammatory microenvironments, achieving effective synergistic interactions among multiple functions remains challenging (Pan Q. et al., 2025). Thus, limited functionality continues to restrict therapeutic effectiveness.

### 7.3 Insufficient control over drug release

Drug release behavior critically influences hydrogel therapeutic efficacy. Many current systems still exhibit initial burst release, characterized by rapid drug release immediately after administration (Xu S. et al., 2023). Conventional hydrogels often struggle to achieve sustained and stable release under complex physiological conditions, as release kinetics largely depend on network structure and the surrounding environment (Li and Mooney, 2016). Such burst release results in excessively high local drug concentrations and reduced therapeutic duration, making it difficult to meet dynamic therapeutic requirements in periodontitis treatment. Consequently, achieving precise and controlled drug release remains a key research focus.

### 7.4 Challenges in clinical translation

Although hydrogel-based therapies for periodontitis have shown promising results in experimental studies, their clinical application remains limited. Most current research is still confined to *in vitro* experiments or small animal models, with relatively limited evidence from large animal studies and clinical trials (Xu S. et al., 2023). In addition, maintaining long-term stability and retention of injectable hydrogels within the oral environment remains challenging, as periodontal pockets are continuously exposed to saliva, bacterial recolonization, and mechanical forces during mastication. The increasing complexity of multifunctional hydrogel systems also introduces additional translational and manufacturing considerations, including ensuring consistent mechanical properties, controlled degradation, and predictable drug release profiles (Dal-Fabbro et al., 2025). Variability in crosslinking behavior and injectability may further affect batch-to-batch reproducibility, while the long-term biosafety of certain nanomaterials and degradation products under chronic oral exposure conditions still requires further investigation. Together, these issues continue to limit the clinical translation of injectable hydrogel systems for periodontitis.

### 7.5 Biocompatibility concerns

Good biocompatibility is fundamental for hydrogels in periodontal applications. Although natural polymers generally demonstrate favorable biocompatibility, performance enhancements often rely on chemical modifications, introducing potential biosafety risks (Li and Mooney, 2016; Liu et al., 2022). Composite hydrogel systems incorporating nanomaterials or functional components can significantly impact cell viability. For example, high concentrations of inorganic nanoparticles in GelMA-based systems may inhibit cell proliferation and compromise biocompatibility (Liu et al., 2022). Moreover, the degradation behavior of hydrogels and the safety of their degradation products remain critical considerations for clinical application.

## 8 Future perspectives and optimization strategies

### 8.1 Multifunctional composite modification

Given the complex etiology of periodontitis, hydrogels with single functionalities are typically inadequate for comprehensive therapy. Therefore, upcoming research should emphasize the development of multifunctional composite hydrogels that integrate antimicrobial, anti-inflammatory, and regenerative properties to achieve synergistic therapeutic outcomes targeting multiple pathological aspects. Frequently adopted strategies involve blending natural polymers with synthetic counterparts or embedding bioactive compounds and functional nanomaterials into hydrogels. A pertinent example includes chitosan/β-glycerophosphate hydrogels embedded with minocycline-loaded ZIF-8 nanoparticles, which concurrently exhibit antibacterial, anti-inflammatory, and tissue regenerative functions (Li et al., 2025d). Another notable instance is the integration of curcumin nanoparticles into chitosan-based antimicrobial peptide hydrogels (Xu S. et al., 2023). Rather than functioning solely as local drug delivery carriers, next-generation injectable hydrogels for periodontitis may increasingly evolve toward multifunctional microenvironment-regulating platforms. Future hydrogel design should integrate antibacterial activity, immunomodulation, oxidative stress regulation, and tissue regenerative functions within a unified therapeutic framework. In this context, dynamically responsive systems capable of adapting to pathological alterations in the periodontal microenvironment may provide more precise and stage-dependent therapeutic regulation, thereby improving the coordination between inflammation control and periodontal regeneration.

### 8.2 Precise regulation of physicochemical properties

Mechanical strength, degradation rate, and drug release kinetics significantly affect hydrogel performance. Thus, precise control of physicochemical properties remains a critical research direction. Adjusting crosslinking density, polymer concentration, and functional group modifications enables fine-tuning of hydrogel structures to match specific stages of tissue repair. Altering methacrylated hyaluronic acid (MHA) concentrations and UV-crosslinking durations significantly influences compressive strength and degradation rates (Hu et al., 2024). Optimizing nanoparticle content or polymer composition further modulates drug release behaviors and structural properties (Li et al., 2025d; Ouyang et al., 2025).

### 8.3 Development of microenvironment-responsive smart hydrogels

Conventional hydrogels relying on passive diffusion are limited in addressing the changing conditions of periodontitis lesions. Microenvironment-responsive hydrogels have therefore attracted attention. In periodontitis, acidic pockets can trigger controlled drug release. The Methotrexate-loaded ZIF-8/chitosan/β-glycerophosphate hydrogel has been shown to release minocycline faster under acidic conditions, allowing localized and sustained delivery within periodontal pockets (Li et al., 2025d). Additionally, ROS-responsive systems can simultaneously provide antibacterial, anti-inflammatory, and immunomodulatory effects (Xu S. et al., 2023; Luo et al., 2024).

### 8.4 Promoting clinical translation and development of advanced materials

Although injectable hydrogels show significant therapeutic potential in animal studies, clinical translation remains limited (Wang S. et al., 2026). Most research still focuses on *in vitro* experiments or small animal models, lacking sufficient validation through large animal studies and clinical trials (Wang S. et al., 2026). Future studies should optimize fabrication processes, establish systematic evaluations from small to large animal models, and perform long-term assessments of biocompatibility and degradation safety (Pan Q. et al., 2025; Wang S. et al., 2026). Concurrently, developing advanced biomaterials will provide additional opportunities for hydrogel optimization (Pan Q. et al., 2025). Biomimetic hydrogel scaffolds combining decellularized bone matrix with GelMA, for example, offer structural mimicry and synergistic delivery of bioactive factors, representing promising strategies for periodontal tissue regeneration. Although injectable hydrogels show significant therapeutic potential in animal studies, clinical translation remains limited (Wang S. et al., 2026). Most research still focuses on *in vitro* experiments or small animal models, lacking sufficient validation through large animal studies and clinical trials (Wang S. et al., 2026). Future studies should optimize fabrication processes, establish systematic evaluations from small to large animal models, and perform long-term assessments of biocompatibility and degradation safety (Pan Q. et al., 2025; Wang S. et al., 2026). Concurrently, developing advanced biomaterials will provide additional opportunities for hydrogel optimization (Pan Q. et al., 2025). Biomimetic hydrogel scaffolds combining decellularized bone matrix with GelMA, for example, offer structural mimicry and synergistic delivery of bioactive factors, representing promising strategies for periodontal tissue regeneration.

## References

[B1] Alghamdi B. Jeon H. H. Ni J. Qiu D. Liu A. Hong J. J. (2023). Osteoimmunology in periodontitis and orthodontic tooth movement. Curr. Osteoporos. Rep. 21 (2), 128–146. 10.1007/s11914-023-00774-x 36862360 PMC10696608

[B2] Amato M. Santonocito S. Viglianisi G. Tatullo M. Isola G. (2022). Impact of oral mesenchymal stem cells applications as a promising therapeutic target in the therapy of periodontal disease. Int. J. Mol. Sci. 23 (21), 13419. 10.3390/ijms232113419 36362206 PMC9658889

[B3] Baddouri L. Hannig M. (2024). Probiotics as an adjunctive therapy in periodontitis treatment—Reality or illusion—A clinical perspective. Npj Biofilms Microbiomes 10 (1), 148. 10.1038/s41522-024-00614-5 39681550 PMC11649906

[B4] Bai X. Peng W. Tang Y. Wang Z. Guo J. Song F. (2024). An NIR-Propelled Janus nanomotor with enhanced ROS-scavenging, immunomodulating and biofilm-eradicating capacity for periodontitis treatment. Bioact. Mat. 41, 271–292. 10.1016/j.bioactmat.2024.07.014 PMC11324457 39149593

[B5] Bashir F. Afzaal A. Shahnaz Gilani M. A. Saleem M. Perveen S. (2024). Eco-friendly development of intrinsically antibacterial and mechanically robust self-healing hydrogels using alginate and oval proteins: advancing periodontitis treatment. Eur. Polym. J. 220, 113423. 10.1016/j.eurpolymj.2024.113423

[B6] Bechir E. S. Bechir F. Suciu M. Bechir A. Nicolau A. C. (2025). Twelve-month follow-up after the treatment of periodontal conditions using scaling and root planning alone vs. laser-assisted new attachment procedure. Diagnostics 15 (14), 1799. 10.3390/diagnostics15141799 40722548 PMC12293740

[B7] Bian Z. Zhang H. Chen Y. Yang Y. Lin Q. Chen X. (2025). Clinical effect of Nd:YAG laser-assisted non-surgical periodontal treatment on stage II/III periodontitis. J. Dent. 162, 106056. 10.1016/j.jdent.2025.106056 40876623

[B8] Botelho J. Lyra P. Nascimento G. G. Leite F. R. M. Mendes J. J. Machado V. (2025). Antibiotics in periodontal treatment: an umbrella review. Front. Cell. Infect. Microbiol. 15, 1601464. 10.3389/fcimb.2025.1601464 40535546 PMC12174147

[B9] Cai G. Ren L. Yu J. Jiang S. Liu G. Wu S. (2024). A microenvironment‐responsive, controlled release hydrogel delivering embelin to promote bone repair of periodontitis via anti‐infection and osteo‐immune modulation. Adv. Sci. 11 (34), 2403786. 10.1002/advs.202403786 PMC11425865 38978324

[B10] Chava V. K. Vedula B. D. (2013). Thermo‐reversible green tea catechin gel for local application in chronic periodontitis: a 4‐week clinical trial. J. Periodontol. 84 (9), 1290–1296. 10.1902/jop.2012.120425 23121459

[B11] Chen N. Ren R. Wei X. Mukundan R. Li G. Xu X. (2021). Thermoresponsive hydrogel-based local delivery of simvastatin for the treatment of periodontitis. Mol. Pharm. 18 (5), 1992–2003. 10.1021/acs.molpharmaceut.0c01196 33754729 PMC8096715

[B12] Chen J. Luo A. Xu M. Zhang Y. Wang Z. Yu S. (2024). The application of phenylboronic acid pinacol ester functionalized ROS-Responsive multifunctional nanoparticles in the treatment of periodontitis. J. Nanobiotechnology 22 (1), 181. 10.1186/s12951-024-02461-0 38622641 PMC11017612

[B13] Chen J. Guan X. Chen L. Zheng B. Li F. Fang C. (2025a). Customized hydrogel system for the spatiotemporal sequential treatment of periodontitis propelled by ZEB1. Adv. Sci. 12 (26), 2503338. 10.1002/advs.202503338 PMC12245124 40184628

[B14] Chen X. Huang H. Guo C. Zhu X. Chen J. Liang J. (2025b). Controlling alveolar bone loss by hydrogel‐based mitigation of oral dysbiosis and bacteria‐triggered proinflammatory immune response. Adv. Funct. Mat. 35 (3), 2409121. 10.1002/adfm.202409121

[B15] Chen Y. Guo B. Zhang Y. Bao X. Li D. Lin J. (2025c). Injectable hypoxia-preconditioned human exfoliated deciduous teeth stem cells encapsulated within GelMA-AMP microspheres for bone regeneration in periodontitis. Colloids Surf. B Biointerfaces 247, 114452. 10.1016/j.colsurfb.2024.114452 39689590

[B16] Chen X. Hu Y. Xu C. Lian T. Li H. Sun L. (2026). Injectable dual-drug hydrogel containing curcumin and glycyrrhizic acid for biofilm inhibition and immunomodulatory therapy in periodontitis. J. Nanobiotechnology 24 (1), 327. 10.1186/s12951-026-04219-2 41772582 PMC13059150

[B17] Chien K.-H. Chang Y.-L. Wang M.-L. Chuang J.-H. Yang Y.-C. Tai M.-C. (2018). Promoting induced pluripotent stem cell-driven biomineralization and periodontal regeneration in rats with maxillary-molar defects using injectable BMP-6 hydrogel. Sci. Rep. 8 (1), 114. 10.1038/s41598-017-18415-6 29311578 PMC5758833

[B18] Da Rocha H. A. J. Silva C. F. Santiago F. L. Martins L. G. Dias P. C. De Magalhães D. (2015). Local drug delivery systems in the treatment of periodontitis: a literature review. J. Int. Acad. Periodontol. 17 (3), 82–90. 26373225

[B19] Dal-Fabbro R. Daghrery A. Anselmi C. Soares I. P. M. Reis-Prado A. H. D. Oliveira P. H. C. de (2025). Recent advances in injectable hydrogel biotherapeutics for regenerative dental medicine. Macromol. Biosci. 25 (10), e00096. 10.1002/mabi.202500096 40605036 PMC12530706

[B20] Dimatteo R. Darling N. J. Segura T. (2018). *In* situ forming injectable hydrogels for drug delivery and wound repair. Adv. Drug Deliv. Rev. 127, 167–184. 10.1016/j.addr.2018.03.007 29567395 PMC6003852

[B21] El-Nablaway M. Rashed F. Taher E. S. Atia G. A. Foda T. Mohammed N. A. (2024). Bioactive injectable mucoadhesive thermosensitive natural polymeric hydrogels for oral bone and periodontal regeneration. Front. Bioeng. Biotechnol. 12, 1384326. 10.3389/fbioe.2024.1384326 38863491 PMC11166210

[B22] El-Sherbiny I. M. Yacoub M. H. (2013). Hydrogel scaffolds for tissue engineering: progress and challenges. Glob. Cardiol. Sci. Pract. 2013 (3), 316–342. 10.5339/gcsp.2013.38 24689032 PMC3963751

[B23] Elgendy H. A. Makky A. M. A. Elakkad Y. E. Ismail R. M. Younes N. F. (2023). Syringeable atorvastatin loaded eugenol enriched PEGylated cubosomes *in-situ* gel for the intra-pocket treatment of periodontitis: statistical optimization and clinical assessment. Drug Deliv. 30 (1), 2162159. 10.1080/10717544.2022.2162159 36604813 PMC9833412

[B24] Fang X. Wang J. Ye C. Lin J. Ran J. Jia Z. (2024). Polyphenol-mediated redox-active hydrogel with H_2_S gaseous-bioelectric coupling for periodontal bone healing in diabetes. Nat. Commun. 15 (1), 9071. 10.1038/s41467-024-53290-6 39433776 PMC11494015

[B25] Ge X. Hu J. Qi X. Shi Y. Chen X. Xiang Y. (2025a). An immunomodulatory hydrogel featuring antibacterial and reactive oxygen species scavenging properties for treating periodontitis in diabetes. Adv. Mat. 37 (3), 2412240. 10.1002/adma.202412240 39610168

[B26] Ge X. Zeng F. Chen Q. Zhao Z. Xie H. Wu G. (2025b). Inflammation-responsive biodegradable nanocomposite hydrogels for enhanced metalloimmunotherapy in chronic periodontitis. Acta Biomater. 208, 293–308. 10.1016/j.actbio.2025.10.058 41176041

[B27] Gui Y. Zhang Y. Xu H. Yang W. Huang H. Gu M. (2025). Recent advances in hydrogels for treating periodontal diseases and oral mucosal diseases. Front. Bioeng. Biotechnol. 13, 1605672. 10.3389/fbioe.2025.1605672 40831626 PMC12358363

[B28] Hajishengallis G. Chavakis T. Lambris J. D. (2020). Current understanding of periodontal disease pathogenesis and targets for host‐modulation therapy. Periodontol. 2000 84 (1), 14–34. 10.1111/prd.12331 32844416 PMC7457922

[B29] Harsas N. A. Bachtiar E. W. Amir L. R. Mauludin R. Sunarso S. Yosefa V. (2025). Bone graft paste nanohydroxyapatite chitosan-gelatin (nHA/KG) for periodontal regeneration: study on three-dimensional cell culture. Eur. J. Dent. 19 (4), 1035–1045. 10.1055/s-0044-1800826 40073987 PMC12494428

[B30] Hienz S. A. Paliwal S. Ivanovski S. (2015). Mechanisms of bone resorption in periodontitis. J. Immunol. Res. 2015, 615486. 10.1155/2015/615486 26065002 PMC4433701

[B31] Hou Y. Li Y. Yang A. Lu X. Han X. Yang Z. (2025). Revolutionizing periodontitis treatment: the promise of GelMA hydrogel. Int. J. Pharm. 681, 125850. 10.1016/j.ijpharm.2025.125850 40523544

[B32] Hu K. Olsen B. R. (2016). Osteoblast-derived VEGF regulates osteoblast differentiation and bone formation during bone repair. J. Clin. Invest. 126 (2), 509–526. 10.1172/JCI82585 26731472 PMC4731163

[B33] Hu Z. Lv X. Zhang H. Zhuang S. Zheng K. Zhou T. (2024). An injectable gel based on photo-cross-linkable hyaluronic acid and mesoporous bioactive glass nanoparticles for periodontitis treatment. Int. J. Biol. Macromol. 257, 128596. 10.1016/j.ijbiomac.2023.128596 38052282

[B34] Hu Y. Xu W. Sun L. Ma X. Zhou P. Zhang C. (2025). Multifunctional injectable hydrogel incorporating EGCG-Cu complexes for synergistic antibacterial, immunomodulatory, and osteogenic therapy in periodontitis. Mat. Today Bio 32, 101907. 10.1016/j.mtbio.2025.101907 PMC12164233 40520560

[B35] Huang R.-L. Yuan Y. Zou G.-M. Liu G. Tu J. Li Q. (2014). LPS-Stimulated inflammatory environment inhibits BMP-2-induced osteoblastic differentiation through crosstalk between TLR4/MyD88/NF-κB and BMP/smad signaling. Stem Cells Dev. 23 (3), 277–289. 10.1089/scd.2013.0345 24050190 PMC3904516

[B36] Huang Y. Wu J. Zhan C. Liu R. Zhou Z. Huang X. (2023). TRAF-STOP alleviates osteoclastogenesis in periodontitis. Front. Pharmacol. 14, 1119847. 10.3389/fphar.2023.1119847 37261283 PMC10229065

[B37] Ivanov A. A. Kuznetsova A. V. Popova O. P. Danilova T. I. Latyshev A. V. Yanushevich O. O. (2023). Influence of extracellular matrix components on the differentiation of periodontal ligament stem cells in collagen I hydrogel. Cells 12 (19), 2335. 10.3390/cells12192335 37830549 PMC10571948

[B38] Jiang W. Ou Y. Yan M. Lu J. Yuan W. He K. (2026). Bindarit-loaded hydrogel sustained-release system alleviates periodontitis in mice via C C motif chemokine ligand 2-dependent monocyte chemotaxis. J. Control. Release 389, 114451. 10.1016/j.jconrel.2025.114451 41276185

[B39] Joseph P. Prabhakar P. Holtfreter B. Pink C. Suvan J. Kocher T. (2023). Systematic review and meta-analysis of randomized controlled trials evaluating the efficacy of non-surgical periodontal treatment in patients with concurrent systemic conditions. Clin. Oral Investig. 28 (1), 21. 10.1007/s00784-023-05392-6 PMC10751251 38147183

[B40] Khademi R. Hosseini M. A. Kharaziha M. (2025). An injectable gelatin methacrylate containing surface-imprinted chitosan-modified bioglass microspheres for potential periodontitis treatment. Int. J. Biol. Macromol. 302, 140561. 10.1016/j.ijbiomac.2025.140561 39894129

[B41] Khodaverdi E. Tekie F. S. M. Mohajeri S. A. Ganji F. Zohuri G. Hadizadeh F. (2012). Preparation and investigation of sustained drug delivery systems using an injectable, thermosensitive, in situ forming hydrogel composed of PLGA–PEG–PLGA. AAPS PharmSciTech 13 (2), 590–600. 10.1208/s12249-012-9781-8 22528547 PMC3364385

[B42] Li J. Mooney D. J. (2016). Designing hydrogels for controlled drug delivery. Nat. Rev. Mat. 1 (12), 16071. 10.1038/natrevmats.2016.71 29657852 PMC5898614

[B43] Li J. Li M. Zhang C. Fei Y. Wang Y. Zhong Z. (2024a). Active targeting microemulsion-based thermosensitive hydrogel against periodontitis by reconstructing Th17/Treg homeostasis via regulating ROS-Macrophages polarization cascade. Int. J. Pharm. 659, 124263. 10.1016/j.ijpharm.2024.124263 38815639

[B44] Li Q. Wang D. Xiao C. Wang H. Dong S. (2024b). Advances in hydrogels for periodontitis treatment. ACS Biomater. Sci. Eng. 10 (5), 2742–2761. 10.1021/acsbiomaterials.4c00220 38639082

[B45] Li C. Yue X. Gao H. Meng Q. Qu J. Chen Y. (2025a). Injectable composite hydrogels based on minocycline and NIR photothermal therapy for antimicrobial and bone regeneration. Biomater. Adv. 177, 214397. 10.1016/j.bioadv.2025.214397 40644855

[B46] Li L. Qin W. Ye T. Wang C. Qin Z. Ma Y. (2025b). Bioactive zn–v–si–ca glass nanoparticle hydrogel microneedles with antimicrobial and antioxidant properties for bone regeneration in diabetic periodontitis. ACS Nano 19 (8), 7981–7995. 10.1021/acsnano.4c15227 39960072

[B47] Li X. Zhang Z. Xie J. Cao B. Wang X. Yu Y. (2025c). A smart injectable hydrogel with dual responsivity to arginine gingipain A and reactive oxygen species for multifunctional therapy of periodontitis. Small 21 (23), 2408034. 10.1002/smll.202408034 40272094

[B48] Li X. Zhang Z. Yang X. Yu M. Tang Y. Wei J. (2025d). Injectable responsive hydrogel with synergistic antibacterial and anti-inflammatory properties for enhanced periodontitis treatment. J. Nanobiotechnology 23 (1), 611. 10.1186/s12951-025-03698-z 41013665 PMC12465922

[B49] Li J. Hou M. Wang R. Li M. Chen J. Ge M. (2026a). Non-antibiotic lipid complex-in-thermogel strikes twice: multimodal photosensitive antibacterial meets immunomodulation-boosted healing for periodontitis treatment. Mat. Today Bio 37, 102781. 10.1016/j.mtbio.2026.102781 PMC12828598 41585442

[B50] Li J. Li R. Zhou Y. Zheng S. Xu J. Zhu J. (2026b). Injectable schiff base-engineered hydrogel for spatiotemporal liraglutide delivery orchestrates diabetic periodontitis regression via multimodal microenvironment reprogramming. J. Control. Release 392, 114688. 10.1016/j.jconrel.2026.114688 41651380

[B51] Lin J. He Z. Liu F. Feng J. Huang C. Sun X. (2020). Hybrid hydrogels for synergistic periodontal antibacterial treatment with sustained drug release and NIR-responsive photothermal effect. Int. J. Nanomedicine 15, 5377–5387. 10.2147/IJN.S248538 32848384 PMC7425099

[B52] Liu M. Zeng X. Ma C. Yi H. Ali Z. Mou X. (2017). Injectable hydrogels for cartilage and bone tissue engineering. Bone Res. 5 (1), 17014. 10.1038/boneres.2017.14 28584674 PMC5448314

[B53] Liu S. Wang Y.-N. Ma B. Shao J. Liu H. Ge S. (2021). Gingipain-responsive thermosensitive hydrogel loaded with SDF-1 facilitates in situ periodontal tissue regeneration. ACS Appl. Mat. Interfaces 13 (31), 36880–36893. 10.1021/acsami.1c08855 34324286

[B54] Liu Y. Li T. Sun M. Cheng Z. Jia W. Jiao K. (2022). ZIF-8 modified multifunctional injectable photopolymerizable GelMA hydrogel for the treatment of periodontitis. Acta Biomater. 146, 37–48. 10.1016/j.actbio.2022.03.046 35364317

[B55] Liu X. Wan X. Sui B. Hu Q. Liu Z. Ding T. (2024a). Piezoelectric hydrogel for treatment of periodontitis through bioenergetic activation. Bioact. Mat. 35, 346–361. 10.1016/j.bioactmat.2024.02.011 PMC10876489 38379699

[B56] Liu Y. Wei X. Yang T. Wang X. Li T. Sun M. (2024b). Hyaluronic acid methacrylate/Pluronic F127 hydrogel enhanced with spermidine-modified mesoporous polydopamine nanoparticles for efficient synergistic periodontitis treatment. Int. J. Biol. Macromol. 281, 136085. 10.1016/j.ijbiomac.2024.136085 39353520

[B57] Liu Y. Yan J. Chen L. Liao Y. Huang L. Tan J. (2024c). Multifunctionalized and dual‐crosslinked hydrogel promotes inflammation resolution and bone regeneration via NLRP3 inhibition in periodontitis. Small Struct. 5 (3), 2300281. 10.1002/sstr.202300281

[B58] Luo Q. Yang Y. Ho C. Li Z. Chiu W. Li A. (2024). Dynamic hydrogel–metal–organic framework system promotes bone regeneration in periodontitis through controlled drug delivery. J. Nanobiotechnology 22 (1), 287. 10.1186/s12951-024-02555-9 38797862 PMC11129436

[B59] Luo A. Yao Y. Chen Y. Li Z. Wang X. (2025). Gelatin methacryloyl-phenylboronic acid/hydroxyadamantane self-healing microgels for the periodontitis treatment by promoting alveolar bone regeneration. Int. J. Biol. Macromol. 303, 140434. 10.1016/j.ijbiomac.2025.140434 39884616

[B60] Ma X. Sekhar K. P. C. Zhang P. Cui J. (2024). Advances in stimuli-responsive injectable hydrogels for biomedical applications. Biomater. Sci. 12 (21), 5468–5480. 10.1039/D4BM00956H 39373614

[B61] Ma G. Xu K. Yu L. Haag R. (2025a). pH‐responsive polyglycerol nanogels for periodontitis treatment through antibacterial and pro‐angiogenesis action. Angew. Chem. Int. Ed. 64 (9), e202418882. 10.1002/anie.202418882 39828663

[B62] Ma S. Yan Q. Li L. Ni Y. Chen K. Xu B. (2025b). Photosensitive small intestinal submucosal hydrogels loaded with the KR-12-a5 peptide promote periodontal osteogenesis and antimicrobial activity. J. Nanobiotechnology 23 (1), 637. 10.1186/s12951-025-03601-w 41074027 PMC12514852

[B63] Mallamma T. Rehman S. A. Goudanavar P. Akondi B. R. (2024). *In* situ gels for periodontitis: an overview. Asian J. Pharm. Res. Health Care 16 (3), 245–252. 10.4103/ajprhc.ajprhc_86_24

[B64] Mao C.-Y. Wang Y.-G. Zhang X. Zheng X.-Y. Tang T.-T. Lu E.-Y. (2016). Double-edged-sword effect of IL-1β on the osteogenesis of periodontal ligament stem cells via crosstalk between the NF-κB, MAPK and BMP/Smad signaling pathways. Cell Death Dis. 7 (7), e2296. 10.1038/cddis.2016.204 27415426 PMC4973347

[B65] Ming P. Li B. Li Q. Yuan L. Jiang X. Liu Y. (2025). Multifunctional sericin-based biomineralized nanoplatforms with immunomodulatory and angio/osteo-genic activity for accelerated bone regeneration in periodontitis. Biomaterials 314, 122885. 10.1016/j.biomaterials.2024.122885 39423514

[B66] Nakajima M. Yanagawa M. Takikawa H. Thien T. T. Zegarra-Caceres L. Yan C. (2025). Advances in local drug delivery for periodontal treatment: present strategies and future directions. Biomolecules 15 (6), 903. 10.3390/biom15060903 40563543 PMC12191426

[B67] Ouyang M. Yu X. Zhong J. Mao Y. Cui Y. Jiang H. (2025). Injectable sodium alginate/4-arm polyethylene glycol-lipoic acid double-network hydrogel loading Ca^2+^-tannic acid nanocomposite treats periodontitis via anti-bacteria, ROS scavenging and osteogenesis. Int. J. Biol. Macromol. 307, 141841. 10.1016/j.ijbiomac.2025.141841 40064278

[B68] Pan P. Liang Q. Xu J. Huang C. Shi J. Zhao L. (2025a). Tetrahydroxy diboron-initiated injectable hydrogel with integrated rapid gelation, fatigue resistance, bioadhesion, antibacterial activity, ROS scavenging, and osteoinduction for periodontitis treatment. Biomacromolecules 26 (10), 6927–6939. 10.1021/acs.biomac.5c01232 40985928

[B69] Pan Q. Zong Z. Li H. Xie L. Zhu H. Wu D. (2025b). Hydrogel design and applications for periodontitis therapy: a review. Int. J. Biol. Macromol. 284, 137893. 10.1016/j.ijbiomac.2024.137893 39571840

[B70] Peng C. Wang G. Li J. Wang Y. Shu Z. Tang M. (2024a). ROS-Responsive and scavenging bifunctional hydrogel enables co-delivery of anti-inflammatory agent and osteogenetic nanoparticle for periodontitis treatment. Mat. Des. 239, 112777. 10.1016/j.matdes.2024.112777

[B71] Peng S. Fu H. Li R. Li H. Wang S. Li B. (2024b). A new direction in periodontitis treatment: biomaterial-mediated macrophage immunotherapy. J. Nanobiotechnology 22 (1), 359. 10.1186/s12951-024-02592-4 38907216 PMC11193307

[B72] Peng Y. Jiang Z. Xu S. He L. Jiang T. Yang Y. (2026). Designing adhesive hydrogels for oral diseases treatment. Mat. Today Bio 37, 102911. 10.1016/j.mtbio.2026.102911 PMC12955167 41782996

[B73] Quan Y. Shao H. Wang N. Gao Z. Jin M. (2025). Microenvironment-sensitive hydrogels as promising drug delivery systems for co-encapsulating microbial homeostasis probiotics and anti-inflammatory drugs to treat periodontitis. Mat. Today Bio 32, 101711. 10.1016/j.mtbio.2025.101711 PMC11994392 40230648

[B74] Ran S. Xue L. Wei X. Huang J. Yan X. He T.-C. (2024). Recent advances in injectable hydrogel therapies for periodontitis. J. Mat. Chem. B 12 (25), 6005–6032. 10.1039/D3TB03070A 38869470

[B75] Rattanasuwan K. Rassameemasmaung S. Sangalungkarn V. Komoltri C. (2016). Clinical effect of locally delivered gel containing green tea extract as an adjunct to non-surgical periodontal treatment. Odontology 104 (1), 89–97. 10.1007/s10266-014-0190-1 25523604

[B76] Ravishankar P. L. Kumar Y. P. Anila E. N. Chakraborty P. Malakar M. Mahalakshmi R. (2017). Effect of local application of curcumin and ornidazole gel in chronic periodontitis patients. Int. J. Pharm. Investig. 7 (4), 188–192. 10.4103/jphi.JPHI_82_17 29692978 PMC5903023

[B77] Shan Y. Zhong J. Sun Q. Gao W. Zhang C. Chen H. (2025). Dual nanozymes-loaded core-shell microneedle patches with antibacterial and NETs-degradation bifunctional properties for periodontitis treatment. Bioact. Mat. 53, 161–177. 10.1016/j.bioactmat.2025.07.003 PMC12274864 40688015

[B78] Shin S.-J. Shim G.-Y. Moon S.-H. Kim Y.-J. Kim H.-J. Oh S. (2025). Injectable thermosensitive hydrogel containing bakuchiol reduces periodontal inflammation and alveolar bone loss in a rat model. J. Funct. Biomater. 16 (8), 292. 10.3390/jfb16080292 40863312 PMC12387751

[B79] Shirbhate U. Bajaj P. (2022). Injectable and self-invigorating hydrogel applications in dentistry and periodontal regeneration: a literature review. Cureus 14 (9), e29248. 10.7759/cureus.29248 36277588 PMC9578657

[B80] Su K. Yan Y. Huang J. Chen Y. Shang X. Wang X. (2026). Mitochondrial-targeted injectable hydrogel for periodontitis therapy via oral immunity and flora regulation. Bioact. Mat. 58, 348–369. 10.1016/j.bioactmat.2025.12.002 PMC12765346 41492366

[B81] Tan N. Sabalic-Schoener M. Nguyen L. D’Aiuto F. (2023). β-Tricalcium phosphate-loaded chitosan-based thermosensitive hydrogel for periodontal regeneration. Polymers 15 (20), 4146. 10.3390/polym15204146 37896389 PMC10611029

[B82] Tao J. Sun Y. Wang G. Sun J. Dong S. Ding J. (2025). Advanced biomaterials for targeting mature biofilms in periodontitis therapy. Bioact. Mat. 48, 474–492. 10.1016/j.bioactmat.2025.02.026 PMC11910363 40093304

[B83] Toledano-Osorio M. Osorio R. Bueno J. Vallecillo C. Vallecillo-Rivas M. Sanz M. (2024). Next‐generation antibacterial nanopolymers for treating oral chronic inflammatory diseases of bacterial origin. Int. Endod. J. 57 (7), 787–803. 10.1111/iej.14040 38340038

[B84] Verma A. Gupta R. Pandit N. Aggarwal S. (2008). Comparative evaluation of subgingivally delivered 10% doxycycline hyclate and xanthan-based chlorhexidine gels in the treatment of chronic periodontitis. J. Contemp. Dent. Pract. 9 (7), 25–32. 10.5005/jcdp-9-7-25 18997913

[B85] Wang G. Wang Y. Ding Y. Chen X. Li S. Zhou W. (2025a). Smart gated hollow mesoporous silica hydrogel for targeting endoplasmic reticulum stress and promoting periodontal tissue regeneration. Adv. Sci. 12 (43), e08400. 10.1002/advs.202508400 PMC12631824 40859424

[B86] Wang H. Wang Y. Yi J. Bai Y. Zhang Y. Hu M. (2025b). Hydrogel loaded with mesalazine for local injected treatment of periodontitis with superior anti-inflammatory, mucosal healing and bone repair performance. Colloids Surf. B Biointerfaces 255, 114881. 10.1016/j.colsurfb.2025.114881 40544687

[B87] Wang J. Li K. Yuan H. (2025c). Preparation of Ag-metal organic frameworks-loaded sodium alginate hydrogel for the treatment of periodontitis. Sci. Rep. 15 (1), 800. 10.1038/s41598-025-85123-x 39755826 PMC11700186

[B88] Wang S. Ren F. Ren L. Zhang J. Song W. Zhang Q. (2026a). Advancing periodontitis treatment with hydrogels: mechanisms and future trends in polymer design and material synergy. Eur. Polym. J. 243, 114502. 10.1016/j.eurpolymj.2026.114502

[B89] Wang X. Ban C. Li X. Wang J. Cao B. Wu W. (2026b). Bioinspired lipid droplets nanoplatform for periodontitis therapy: integrated antibacterial, mitochondrial repair, and immunomodulatory functions. Mat. Today Bio 37, 102808. 10.1016/j.mtbio.2026.102808 PMC12858359 41624519

[B90] Xi Y. Wang Y. Gao J. Xiao Y. Du J. (2019). Dual corona vesicles with intrinsic antibacterial and enhanced antibiotic delivery capabilities for effective treatment of biofilm-induced periodontitis. ACS Nano 13 (12), 13645–13657. 10.1021/acsnano.9b03237 31585041

[B91] Xia Z. Zhao B. Xiang J. Xu K. Luo K. Yu L.-X. (2025). Injectable pH-responsive hydrogel adapted to gingival crevicular fluid microenvironment for periodontitis therapy. ACS Appl. Mat. Interfaces 17 (21), 31357–31367. 10.1021/acsami.5c02776 40383914

[B92] Xiu Z. Zhu Y. Li X. Jiang X. Feng Y. He L. (2025). A multifunctional injectable microsphere with enhanced near-infrared photo-antibacterial, ROS scavenging, and anti-inflammatory properties for periodontitis treatment. Theranostics 15 (9), 3750–3780. 10.7150/thno.107793 40213673 PMC11980662

[B93] Xu S. Hu B. Dong T. Chen B.-Y. Xiong X.-J. Du L.-J. (2023a). Alleviate periodontitis and its comorbidity hypertension using a nanoparticle‐embedded functional hydrogel system. Adv. Healthc. Mat. 12 (20), 2203337. 10.1002/adhm.202203337 36972711

[B94] Xu Y. Luo Y. Weng Z. Xu H. Zhang W. Li Q. (2023b). Microenvironment-responsive metal-phenolic nanozyme release platform with antibacterial, ROS scavenging, and osteogenesis for periodontitis. ACS Nano 17 (19), 18732–18746. 10.1021/acsnano.3c01940 37768714

[B95] Yang S. Zhu Y. Ji C. Zhu H. Lao A. Zhao R. (2024a). A five-in-one novel MOF-Modified injectable hydrogel with thermo-sensitive and adhesive properties for promoting alveolar bone repair in periodontitis: antibacterial, hemostasis, immune reprogramming, pro-osteo-/angiogenesis and recruitment. Bioact. Mat. 41, 239–256. 10.1016/j.bioactmat.2024.07.016 PMC11324614 39149594

[B96] Yang S.-Y. Hu Y. Zhao R. Zhou Y.-N. Zhuang Y. Zhu Y. (2024b). Quercetin-loaded mesoporous nano-delivery system remodels osteoimmune microenvironment to regenerate alveolar bone in periodontitis via the miR-21a-5p/PDCD4/NF-κB pathway. J. Nanobiotechnology 22 (1), 94. 10.1186/s12951-024-02352-4 38449005 PMC10918894

[B97] Yang B. Xu J. Liu S. Wu C. Li Y. Lv M. (2025). Applications of bioactive herbal extracts in dressing materials for skin wound repair: ingredients, mechanisms and innovations. Interdiscip. Med. 3 (4), e20240117. 10.1002/INMD.20240117

[B98] Yao W. Xu P. Pang Z. Zhao J. Chai Z. Li X. (2014). Local delivery of minocycline-loaded PEG-PLA nanoparticles for the enhanced treatment of periodontitis in dogs. Int. J. Nanomedicine 9, 3963–3970. 10.2147/IJN.S67521 25170266 PMC4145825

[B99] Yin B. Dodda J. M. Wong S. H. D. Roshan Deen G. Bate J. S. Pachauri A. (2025). Smart injectable hydrogels for periodontal regeneration: recent advancements in biomaterials and biofabrication strategies. Mat. Today Bio 32, 101855. 10.1016/j.mtbio.2025.101855 PMC12145717 40487163

[B100] You Z. Y. Wu Y. L. Sun Y. M. Wang Z. M. Ye L. (2023). Application of gelatin methacryloyl/minocycline-chitosan-nanoparticles composite hydrogel for the treatment of periodontitis. West China J. Stomatol. 41 (1), 11–20. 10.7518/hxkq.2023.01.002 PMC9988448 38596936

[B101] Yu Y. You Z. Li X. Lou F. Xiong D. Ye L. (2024). Injectable nanocomposite hydrogels with strong antibacterial, osteoinductive, and ROS-Scavenging capabilities for periodontitis treatment. ACS Appl. Mat. Interfaces 16 (12), 14421–14433. 10.1021/acsami.3c16577 38497587

[B102] Yu Q. Zheng Z. Zhang H. Xie E. Chen L. Jiang Z. (2025). Effects of reactive oxygen species and antioxidant strategies on wound healing in diabetes. Interdiscip. Med. 3 (2), e20240062. 10.1002/INMD.20240062

[B103] Yu J. Tang J. Li J. Peng W.-H. Wang G. Lv X. (2026a). Interpenetrating polymer network hydrogels with EGCG-MPN and nitric oxide for periodontitis. J. Mat. Chem. B 14 (15), 4624–4634. 10.1039/D5TB02717A 41914982

[B104] Yu X. Liu H. Chen L. Cheng X. Wu G. Forouzanfar T. (2026b). Thermosensitive antibacterial nanocomposite hydrogel guiding macrophage polarization and bone regeneration for periodontitis treatment. Bioact. Mat. 55, 376–390. 10.1016/j.bioactmat.2025.09.030 PMC12506435 41069762

[B105] Yue K. Trujillo-de Santiago G. Alvarez M. M. Tamayol A. Annabi N. Khademhosseini A. (2015). Synthesis, properties, and biomedical applications of gelatin methacryloyl (GelMA) hydrogels. Biomaterials 73, 254–271. 10.1016/j.biomaterials.2015.08.045 26414409 PMC4610009

[B106] Zhang S. Li X. Li C. Cong H. Yu B. (2024). Research progress of injectable hydrogels in the treatment of bone tissue diseases. Chem. Eng. J. 498, 155139. 10.1016/j.cej.2024.155139

[B107] Zhang J. Meng L. Jia Y. Li J. Xu X. Xu X. (2025). Development of an injectable salicylic acid–choline eutectic hydrogel for enhanced treatment of periodontitis. Mat. Horiz. 12 (11), 3788–3802. 10.1039/D4MH01563K 40052257

[B108] Zhang L. Wang W. Tan H. Zou Q. Yu J. Jia B. (2026). Ligand‐modulated manganese ferrite nanozyme hydrogel with balanced antioxidant activity and biocompatibility for periodontitis treatment. Adv. Healthc. Mat. 15 (15), e05568. 10.1002/adhm.202505568 41622691

[B109] Zhao Z. Wu C. Huangfu Y. Zhang Y. Zhang J. Huang P. (2024). Bioinspired glycopeptide hydrogel reestablishing bone homeostasis through mediating osteoclasts and osteogenesis in periodontitis treatment. ACS Nano 18 (43), 29507–29521. 10.1021/acsnano.4c05677 39401162

[B110] Zhao W. Zhang Y. Chen J. Hu D. (2025). Revolutionizing oral care: reactive oxygen species (ROS)-Regulating biomaterials for combating infection and inflammation. Redox Biol. 79, 103451. 10.1016/j.redox.2024.103451 39631247 PMC11664010

[B111] Zhou H. He Z. Cao Y. Chu L. Liang B. Yu K. (2024a). An injectable magnesium-loaded hydrogel releases hydrogen to promote osteoporotic bone repair via ROS scavenging and immunomodulation. Theranostics 14 (9), 3739–3759. 10.7150/thno.97412 38948054 PMC11209720

[B112] Zhou H. Zhang Y.-F. Zhang Q.-Q. Liu F. Zhang J.-Y. Chen Y. (2024b). Cathepsin K inhibition alleviates periodontal bone resorption by promoting type H vessel formation through PDGF‐BB/PDGFR ‐β axis. Oral Dis. 30 (8), 5335–5348. 10.1111/odi.14920 38462960

[B113] Zhou C.-H. Gao Y. Zhou X.-L. Pan D.-W. Liu Z. Wang W. (2026). Injectable dual-cross-linked hydrogels enabling sequential drug release for synergistic periodontitis therapy. ACS Appl. Mat. Interfaces 18 (13), 18838–18853. 10.1021/acsami.6c01751 41907007

[B114] Zhu S. Li Y. He Z. Ji L. Zhang W. Tong Y. (2022). Advanced injectable hydrogels for cartilage tissue engineering. Front. Bioeng. Biotechnol. 10, 954501. 10.3389/fbioe.2022.954501 36159703 PMC9493100

[B115] Zhu Y. Xiu Z. Jiang X. Zhang H. Li X. Feng Y. (2025). Injectable hydrogels with ROS-Triggered drug release enable the co-delivery of antibacterial agent and anti-inflammatory nanoparticle for periodontitis treatment. J. Nanobiotechnology 23 (1), 205. 10.1186/s12951-025-03275-4 40075491 PMC11900060

[B116] Zubair S. (2025). Targeted use of antimicrobials in periodontal therapy. Cureus 17 (3), e79874. 10.7759/cureus.79874 40171348 PMC11958838

[B117] Zussman M. Zilberman M. (2022). Injectable metronidazole-eluting gelatin-alginate hydrogels for local treatment of periodontitis. J. Biomater. Appl. 37 (1), 166–179. 10.1177/08853282221079458 35341363

